# A Data-Driven Approach for Comparing Gaze Allocation Across Conditions

**DOI:** 10.3390/jemr19020033

**Published:** 2026-03-18

**Authors:** Jack Prosser, Anna Metzger, Matteo Toscani

**Affiliations:** School of Psychology, Bournemouth University, Poole BH12 5BB, UK; psychology@j-prosser.com (J.P.); ametzger@bournemouth.ac.uk (A.M.)

**Keywords:** saliency, data-driven analysis, cross-modal attention

## Abstract

Gaze analysis often relies on hypothesised, subjectively defined regions of interest (ROIs) or heatmaps: ROIs enable condition comparisons but reduce objectivity and exploration; while heatmaps avoid this, they require many pixel-wise comparisons, making differences hard to detect. Here, we propose an advanced data-driven approach for analysing gaze behaviour. We use DNNs (adapted versions of AlexNet) to classify conditions from gaze patterns, paired with reverse correlation to show where and how gaze differs between conditions. We test our approach on data from an experiment investigating the effects of object-specific sounds (e.g., church bell ringing) on gaze allocation. ROI-based analysis shows a significant difference between conditions (congruent sound, no sound, phase-scrambled sound and pink noise), with more gaze allocation on sound-associated objects in the congruent sound condition. However, as expected, significance depends on the definition of the ROIs. Heatmaps show some unclear qualitative differences, but none are significant after correcting for pixelwise comparisons. We showed that, for some scenes, the DNNs could classify the task based on individual fixations with accuracy significantly higher than chance. Our approach shows that sound can alter gaze allocation, revealing task-specific, non-trivial strategies: fixations are not always drawn to the sound source but shift away from salient features, sometimes falling between salient features and the sound source. Crucially, such fixation strategies could not be revealed using a traditional hypothesis-driven approach. Overall, the method is objective, data-driven, and enables clear comparisons of conditions.

## 1. Introduction

Perception is inherently active. Within the visual system, high-resolution vision is limited to the fovea. As such, we move our eyes to bring relevant parts of a scene into focus, selecting them for further processing [1,2]. Saliency refers to the potential of visual stimulation to attract gaze. Early research suggested that saliency is primarily driven by bottom-up image properties such as intensity, contrast, motion and Gestalt properties, computed in a pre-attentive manner and independent of the task at hand [2,3,4]. However, a seminal study by Yarbus demonstrated that gaze behaviour can be influenced by cognitive tasks [5]. Later research has broadened the range of higher-level, top-down factors affecting saliency. For instance, it was shown that task demands and value strongly influence gaze [6,7,8]. Additionally, the visual system directs gaze to task-relevant features—for example, to colour- or motion-diagnostic regions [9,10,11]—in a dynamic manner [12]. Furthermore, saliency is suggested to depend on different features at different timepoints, with later, higher-latency saccades driven by the task, whilst initial, low-latency features being independent of the task [13].

There are different approaches to investigating gaze behaviour. In previous work, eye movement parameters, such as fixation duration or saccadic amplitude, were compared to investigate differences between conditions [14]. However, spatial information about gaze is typically lost in such approaches or is, at the very least, limited. For instance, Metzger and colleagues [13] created stimuli, dissociating perceptually relevant information in space to differentiate between bottom-up and task-driven saliency. Moreover, Aizenman and colleagues [15] showed that horizontal or vertical spread in fixations was higher when participants performed width or height judgments of virtual objects, respectively. Analysis of coverage with fixations as utilised by Toscani and colleagues [16] can additionally inform us of how distributed our gaze allocation is and whether it is a result of multi-modal object comparisons or uni-modal comparisons. To be able to perform such comparisons, it is necessary to use simplified stimuli created in a hypothesis-driven way. However, it is often interesting to investigate gaze on natural images, as they can reveal patterns of visual attention that are more ecologically valid and closer to real-world perception.

Classically, analysis of eye movements on natural images has focused on comparing gaze parameters (e.g., fixation number) on predefined regions of interest (ROIs), otherwise known as areas of interest. For instance, End and Gamer [17] used ROI analysis to demonstrate that the saliency of social elements in natural images (e.g., presence of people, specifically heads and eyes) was increased when participants were instructed to detect people as fast as possible. The study placed social features amongst other highly salient features that could direct attention away from the task goal. However, this approach has been recently criticised for its multiple limitations [18]. (1) When using natural images, it is not always clear which fixations belong to which object, given insufficient distances between objects or even overlap. (2) The definition of ROI margins is usually subjective, and indeed has the potential to affect the results, as systematically assessed by Orquin and colleagues [19]. (3) To obtain a detailed analysis of gaze allocation, such analyses involve comparison of multiple ROIs, inflating the rate of false positives if not corrected [18,20]. For instance, Antúnez and colleagues [21] ran 105 comparisons between ROIs to investigate the allocation of attention on food labels. (4) Another limitation of this approach is that it requires specific hypotheses about how eye movements are used in a specific task and does not reveal other strategies. For instance, fixations landing outside hypothesised ROIs are usually discarded as irrelevant. However, they might instead reflect a sophisticated strategy to simultaneously inspect several locations on the image by minimising their distance to the fovea, hence achieving the highest possible acuity for multiple locations [22].

Spatial fixation density distributions provide a data-driven way to identify the most salient areas of an image. ROIs can be defined using simple thresholding, selecting regions above a certain fixation frequency [23], or through clustering methods, which group nearby fixations without relying on arbitrary cutoff values [24,25]. While these approaches reliably highlight salient regions, they do not ensure that the resulting ROIs are diagnostic for distinguishing experimental conditions. Therefore, they do not solve the problem of arbitrary ROI selection.

Here, we propose a new data-driven approach for analysing differences between conditions in fixation distributions on natural images. The approach involves training a supervised deep neural network (DNN) to classify the task from fixation distributions. Above-chance classification performance indicates that there are systematic differences between conditions. We then use an image classification approach [26,27,28] to graphically visualise the differences in gaze allocation between experimental conditions. The DNN is trained to classify conditions based on fixation distributions, with random fixations drawn from either condition. These fixation distributions are then classified by the network as belonging to one of the experimental conditions. Averaging across all fixation distributions classified as a particular task reveals the classification image for that task. Differences between the conditions’ classification images allow us to visualise spatial differences in salient elements that are diagnostic of each condition.

We test this approach on fixation data from an eye-tracking experiment in which participants viewed natural images, each containing a sound-emitting object (e.g., church bell). We investigated whether a simultaneously played sound consistent with the sound-emitting object in the image would affect gaze allocation (i.e., attracting it towards this object), as compared to a no-sound condition. There is a body of literature on saliency in the different senses (e.g., [2,29,30]). Multisensory interaction effects have been shown on appearance (e.g., the percept of an ambiguous image was shifted to the object congruent with the simultaneously played sound [31]); precision (e.g., the size of an object can be estimated more precisely when integrating estimates from vision and touch [32]); and search facilitation (e.g., multisensory cues improve visual target detection [33]). However, less is known about how information presented to other senses affects gaze allocation (but please see Toscani and colleagues [16]).

The new method provided results consistent with traditional ROI analysis and heatmap inspection. However, it is purely data-driven and allows for comparison between conditions. Furthermore, as it is not hypothesis-driven, it may provide richer insights into the differences in fixation strategies between different conditions. 

## 2. Materials and Methods

### 2.1. Design

The experiment employed a repeated-measures design comprising two independent variables, *sound* (congruent sound vs no sound) and *image* (10 images), resulting in 20 conditions. Each image was presented five times for 2 s. We used fixation distributions to predict the experimental condition for each image.

### 2.2. Participants

The final sample consisted of 7 Bournemouth University undergraduate students (5 females), aged between 18 and 22 years (M = 21.60, SD = 0.49), recruited through the SONA platform. Initially, 15 participants were recruited; however, 8 participants were excluded from analysis due to incomplete data resulting from study disruptions or repeated difficulties in eye-tracking calibration. This is because we regularly checked the eye-tracking calibration and repeated it if necessary (please see Section 2.4). We excluded calibrations with errors greater than 0.5 degrees of visual angle (dva). After repeated failed calibrations, some of the participants withdrew from the study. All participants were naïve to the specific aim of the study and had normal or corrected-to-normal vision and hearing.

As we wanted to compare our novel DNN-based approach with a classic ROI-based factorial design, the sample size was determined using a G*Power (Version 3.1) analysis [34]. The required sample size for a repeated-measures ANOVA with one group and four measurements, a significance level of *α* = 0.05, a desired power of 0.8, and a large effect size (*η*^2^ = 0.14) is 6 participants. More participants were recruited to ensure sufficient successful experiment completions. The choice of large effect sizes is justified by our previous study on visual saliency, which showed a large effect of task on eye movement allocation [13]. Participants provided informed consent and were compensated for their time with course credits at the rate of 1 credit per hour. The study was approved by the ethics committee of Bournemouth University and conducted in accordance with the 2013 Declaration of Helsinki.

### 2.3. Stimuli

**Figure 1:**
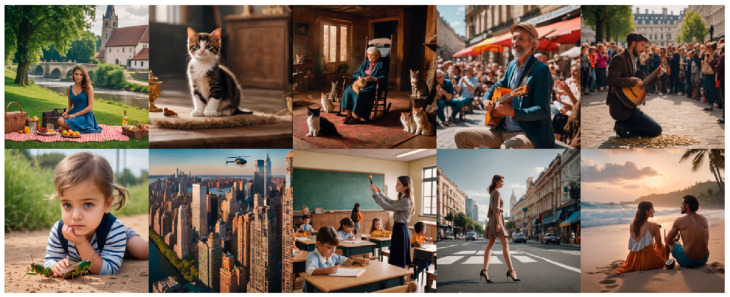
Visual stimuli. Each image depicts an object that produces a sound but is not visually salient. From left to right and top to bottom, the objects are: a bell tower, a bird, a rocking chair, clapping hands, tossing coins, crickets, a helicopter, a school bell, high heels, and the sea. These objects are not presented at the centre of the images, where fixations naturally tend to land.

Ten sound stimuli were selected from publicly available databases [35,36]: church bells chiming, bird tweeting, rocking chair creaking, applause, coins clinking, cicada chirping, helicopter blades beating, bell ringing, heels clacking, waves crashing against the shore. Sounds were cut to two seconds (consistent with the duration of the presentation of the images), containing the most subjectively recognisable part for the target object. For each sound, a phase-scrambled version was created to test if potential effects on gaze can be explained by low-level features of the sound, such as frequency composition. Sound volume was adjusted for each participant so that they could clearly hear and recognise the sounds. It remained constant across the experiment. For each sound, consistent colour images were generated with GenCraft (Image 2.0), https://gencraft.com/, an AI-based software (Figure 1). To be able to investigate the effect of sound on gaze allocation, we tried to exclude the possibility that the sound-emitting object was fixated on because of the salience of its position [37] or its low- or high-level features [2,38] by presenting it alongside other highly salient image features. For example, for the sound of church bells chiming, a scene was designed in which a woman (high salience level) was presented centrally (salient location) at a picnic (low salience level), while the church, being the target object, was presented in the background. Images were presented for 2 s in the middle of the screen on a grey background (rgb = 0.5, 0.5, 0.5).

### 2.4. Setup and Eye-Tracking Recording

Participants sat comfortably at a table in front of a monitor (1920  ×  1080) in a dark room. Head position was controlled via a chinrest. The viewing distance was 58 cm. We used a desktop-mounted eye tracker (EyeLink 1000; SR Research Ltd., Osgoode, ON, Canada) to record gaze position signals sampled at 1000 Hz. The display was viewed binocularly, but only the right eye was tracked. We performed a standard calibration procedure at the beginning of each session [39]. Then, at the beginning of each trial, calibration was checked. If the error exceeded 1.5° of visual angle, recalibration was performed; otherwise, a drift correction was applied. After each calibration, we performed a validation procedure. Calibrations were accepted only if the validation showed a mean error below 0.5 degrees of visual angle (dva), otherwise a new calibration was performed. Eye movements were classified using the standard Eyelink algorithm: acceleration threshold 8000°/s^2^, velocity threshold 30°/s were criteria to detect saccades, consecutive samples without saccades were averaged into a single fixation.

Images were presented on a 24-inch BenQ XL monitor (BenQ Corporation, Taipei, Taiwan) with a resolution of 1920 × 1080 pixels. They were presented in their original sizes in the centre of the screen. We used a standard procedure to colour-calibrate the monitor [40,41], to linearise the screen and make sure that we displayed the desired colour. We measured the gamma curves of each channel and their chromaticity with the Spyder 4 colourimeter (Datacolor, Lawrenceville, NJ, USA). Our screen had the following chromaticity: red primary CIE xyY coordinate (x: 0.6413, y: 0.3274, Y: 57.61 cd/m^2^), green (x: 0.3104, y: 0.6256, Y: 256.98 cd/m^2^), and blue (x: 0.1514, y: 0.0568, Y: 26.2 cd/m^2^). The gamma exponents were 1.913, 1.567 and 2.096, for the red, green and blue channels, respectively.

### 2.5. Procedure

Before each trial, participants were asked to fixate on one of two fixation points (left or right outside of the part of the screen on which the image was presented) to ensure that the first fixation was informative and not biassed by the fixation point between the trials [42]. Once fixated, participants pressed the space bar to initiate the trial. Calibration was checked and drift correction or recalibration was performed when necessary. In each trial, participants viewed, for 2 s, one of ten images, which was either accompanied by a sound or not, depending on the condition. Every 10th image was displayed on a green background, indicating to participants to decide if this image was “old” (i.e., presented among the previous 9) or “new” (right or left arrow respectively). The order of images and conditions was randomised for each participant. Each image in each condition was presented 5 times.

### 2.6. Analysis

#### 2.6.1. ROI Analysis

**Figure 2:**
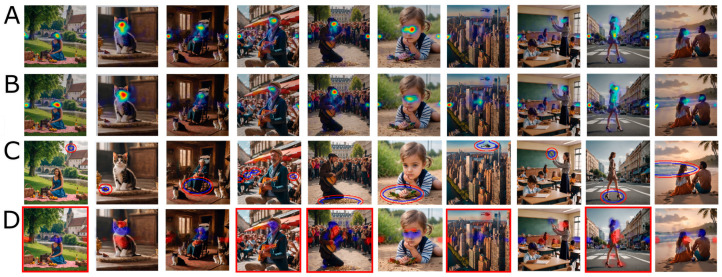
Heatmaps and regions of interest. (**A**) Heatmaps for the consistent-sound condition averaged across participants, and (**B**) heatmaps for the no-sound condition averaged across participants; fixation density increases from no colour to blue, yellow, and red. (**C**) Regions of interest (ROIs), with smaller ROIs indicated by blue ellipses and larger ROIs by red ellipses. (**D**) Heatmaps revealed by classification image analysis, where blue indicates negative differences (i.e., fewer condition-diagnostic fixations in the consistent-sound condition) and red indicates positive differences (i.e., more condition-diagnostic fixations in the consistent-sound condition); colour intensity reflects the absolute magnitude of the difference. Images surrounded by a red square are those for which we could significantly classify the condition based on fixations using our DNN-based approach.

Two 10 (image) × 2 (consistent, no sound) repeated-measures ANOVAs were used to test for differences in frequency of fixations in the ROIs. We manually defined the ROIs as ellipses around the object (Figure 2C). We did not simply segment the objects and used their surface as ROIs because, due to inaccuracies in both eye-tracking and the human visual system, fixations frequently land outside the target object [18].

To evaluate how robust the results are to small variations in the ROI definition, we created both a smaller and a slightly larger version of each ROI. The smaller ROI was obtained by eroding the original elliptical mask using MATLAB’s (R2025b, The MathWorks, Inc., Natick, MA, USA) morphological operations. Specifically, we used the function strel () to create a disc-shaped structuring element with a radius of pixels corresponding to approximately 0.78 dva. This structuring element was then applied to the ROI mask using the function imerode (), which uniformly shrinks the mask boundaries. As a result, the smaller ellipse was approximately 1.6 dva smaller at each peripheral point compared to the original ROI. For each image, we computed the proportion of fixations within the ROI per participant. This was our dependent variable for the ANOVAs. We conducted two ANOVAs, one for the larger and one for the smaller ROIs.

#### 2.6.2. DNN-Based Approach

For each image, we trained a deep neural network (DNN) to classify individual fixations into either the sound or the no-sound condition.

##### Training and Validation Data

To train the DNN, we generated a large number of single-fixation images by randomly sampling real fixations from all participants. Crucially, the training and validation sets were created by sampling fixations from different trials: only one repetition for each image, condition and participant, which was not used for the training was used for validation. When the training and validation sets were sampled from the same fixation distribution, network performance was close to 100% for each image, suggesting that the network had simply learned to represent the fixation distribution associated with each condition. Performance was lower—but still very high—when we used two different sets of fixations to create the training and validation sets while allowing fixations from the same trial to appear in both sets. This is presumably because fixations that are close in time also tend to be close in space. Our decision to split the data by trials was therefore intended to avoid overfitting by ensuring that training and validation were performed on genuinely independent datasets. There might be some differences in gaze between repetitions; however, being trained and validated on different repetitions, the DNN is forced to learn the gaze features that are characteristic to the conditions, abstracting from changes across conditions. We arbitrarily decided to create 180,000 training images and 60,000 validation images. To generate these, each fixation was marked as a value of 1 in a matrix of zeros corresponding to the image size. The resulting image was then Gaussian-filtered with a sigma corresponding to one dva. This was done to approximate the size of the fovea and to account for the resolution of the eye tracker (we accepted calibrations with no more than a 0.5 DVA average error). The images were then resized to 227 × 227 pixels for computational efficiency.

##### DNN

We used AlexNet, a popular and relatively simple DNN architecture for image classification. The only modification to the original architecture was adapting the final layer to output two classes corresponding to the experimental conditions of interest. After piloting, we decided on the following optimisation parameters: training was performed using stochastic gradient descent with momentum (SGDM), with a mini-batch size of 32 and a maximum of 8 epochs. The initial learning rate was set to $6\times 10^{-4}$ and followed a piecewise schedule, decreasing by a factor of 0.1 every 4 epochs. Momentum was fixed at 0.9 and L2 regularisation at $1\times 10^{-4}$. Training data were shuffled at every epoch. Model performance was monitored on a held-out validation set at a frequency of 50 iterations, and training was executed on a GPU within a Linux workstation running Ubuntu 22.04.5 LTS (Canonical Ltd., London, UK), equipped with an AMD Ryzen Threadripper PRO 5955WX CPU (Advanced Micro Devices, Inc., Santa Clara, CA, USA; 16 cores, 32 threads) 256 GB RAM, and an NVIDIA RTX A6000 GPU with 48 GB VRAM (NVIDIA Corporation, Santa Clara, CA, USA). During training, we applied online data augmentation to the fixation images to improve generalisation and reduce overfitting. Augmentations included random rotations (±20°), horizontal and vertical translations (±10 pixels), independent scaling along the x- and y-axes (0.8–1.2), and random horizontal reflections. These transformations preserve the overall structure of the fixation patterns while introducing variability consistent with natural spatial uncertainty.

##### Statistical Inference

**Figure 3:**
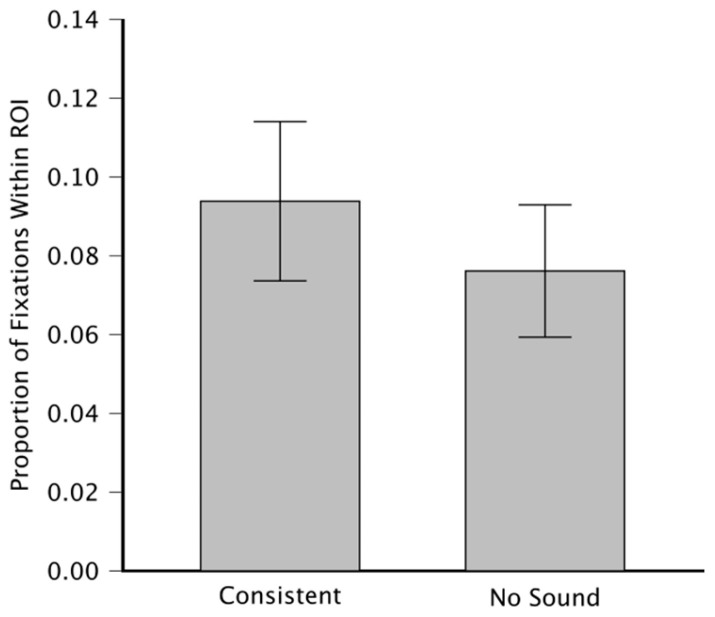
Proportion of fixations within ROIs, on the y-axis, averaged across images and across participants. Consistent-sound and no-sound conditions on the x-axis. Error bars indicate the standard error of the mean across participants.

We performed bootstrap inference to evaluate the reliability of our network classification accuracy. To establish empirical chance levels, each network was first trained with randomly shuffled condition labels for each image, yielding accuracies around 50% (Figure 3). For networks trained on the true labels, we then resampled 1000 validation images with replacement and computed classification accuracy on each resampled set, repeating this process 100 times to build an empirical distribution of accuracy estimates. This strategy—sometimes referred to as bootstrapping the test/validation predictions—uses a trained model’s existing predictions on held-out data and resamples them to estimate the sampling distribution of the performance metric without retraining the network on new bootstrap training sets; it is a computationally efficient way to obtain confidence intervals for classifier performance [43]. To account for multiple comparisons across the 10 images, we applied a Bonferroni correction, resulting in a 99.5% confidence interval. We then assessed whether this interval included the empirical chance level; if it did not, the network’s performance was considered statistically significant.

## 3. Results

Figure 2A,B show heatmaps averaged across participants in the consistent-sound and in the no-sound conditions, respectively.

### 3.1. ROI Analysis

The ANOVA on smaller ROIs (blue ellipses in Figure 2C) shows a significant main effect of sound (F(1,5) = 6.708, *p* = 0.049, $\eta_{p}^{2}$ = 0.56), a main effect of image (F(9,45) = 68.589, *p* < 0.001; $\eta_{p}^{2}$ = 0.93) and no significant interaction (F(9,45) = 0.884, *p* = 0.547; $\eta_{p}^{2}$ = 0.15). This indicates that the proportion of fixations with the ROI is, on average, higher in the consistent-sound condition than in the no-sound condition, as shown in Figure 3.

With this analysis, the main effect of image is expected because of the different size of the ROIs for different images. In fact, we included a blocking factor to account for ROI size differences across images. Crucially, when we repeated the ANOVA for the larger ROIs, the main effect of sound condition was no longer significant (F (1,5) = 4.195, *p* = 0.096, $\eta_{p}^{2}$ = 0.456). This shows how a little difference in the choice of the ROIs may affect the results.

### 3.2. DNN-Based Analysis

We tested whether individual fixations could predict the experimental condition for each of the 10 images. Classification was performed using a separate neural network per image, and accuracy was evaluated on a held-out validation set (see Section 2).

**Figure 4:**
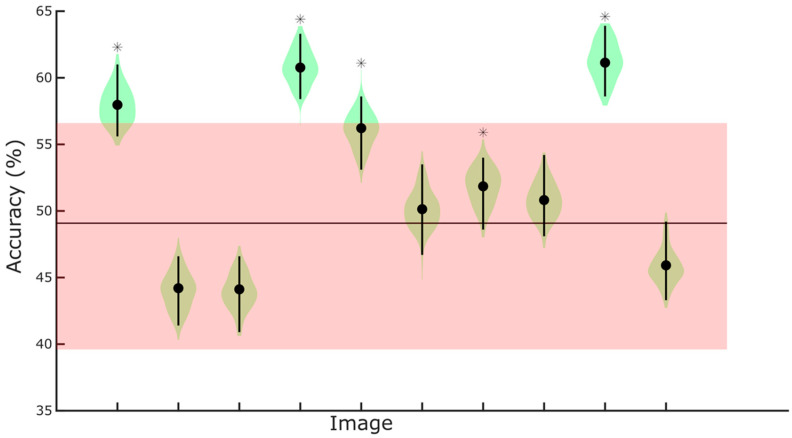
Classification results. Image on the x-axis, accuracy on the y-axis, in percentage. Black circles indicate classification accuracy computed on the whole validation set. The violin plot shaded areas (in green) represent the kernel density estimate of the data, indicating the distribution’s shape and relative frequency. The vertical lines represent the interquartile range. Asterisks indicate the images for which classification was significantly above chance (see Table 1). The horizontal line represents the average empirical chance level, and the shaded (in red) area around it represents its 95% confidence interval.

Figure 4 shows the validation set accuracy alongside bootstrapped distributions for each image. Bootstrapped 99.5% confidence intervals (Bonferroni-corrected; corresponding to the 0.0025 and 0.9975 quantiles) provided an estimate of variability in classification accuracy.

Across images, classification accuracy exceeded chance levels for 5 out of 10 images. For the five images showing significant classification above chance (images 1, 4, 5, 8, and 9), the lower bound of the bootstrap confidence interval exceeded the empirical chance level, indicating that the observed performance was unlikely to occur by chance (see Table 1). For the remaining images, the bootstrap intervals overlapped with chance, suggesting that classification was not reliable for these stimuli.

### 3.3. Classification Images

Figure 2D shows the heatmaps revealed by classification image analysis. These heatmaps visualise the spatial differences in condition-diagnostic fixations between the sound and no-sound conditions. While in some cases, diagnostic fixations increased at locations corresponding to the expected ROI around the sound-emitting object, in several images, the maps reveal more distributed patterns: fixations in the sound condition were reduced on central or otherwise highly salient regions, such as faces, and increased on peripheral or task-relevant areas closer to the sound source. For example, in image 1, participants fixated less on the central face and more on lateral image regions, whereas in image 9, gaze is shifted away from the face toward the body and the sound-emitting shoe. Overall, these visualisations highlight both expected and distributed condition-diagnostic fixation differences that were not consistently captured by traditional ROI analyses.

## 4. Discussion

In this study, participants viewed natural images containing a potential sound-emitting object while listening either to a consistent sound or to no sound. Crucially, the sound-emitting objects were intentionally chosen to be not visually salient and were generally not located at the centre of the scene to avoid simple saliency effects dominating gaze behaviour. We analysed gaze allocation using (1) a classical ROI analysis centred on hypothesised task-relevant regions and (2) a data-driven method that trained a supervised deep neural network (DNN) to classify the task condition from single fixations.

Both methods provided evidence that auditory context modulates gaze behaviour. The ROI analysis showed a significant main effect of sound when fixations fell within predefined spatial regions around the sound-emitting object. However, these effects were highly sensitive to arbitrary ROI definitions: small adjustments to ROI size were sufficient to eliminate statistical significance. In contrast, the DNN classifier reliably decoded the condition from single fixations for several images, demonstrating that systematic differences existed in spatial gaze patterns that were not robustly captured within hand-drawn ROIs. Reverse correlation allows us to visualise the spatial locations that drive these classification decisions.

ROI-based analysis has yielded important results in eye-movement research, especially in hypothesis-driven studies, where predictions about feature-specific fixations—such as expression-specific diagnostic face regions [44]—can be directly tested. However, the limitations of ROI-based analysis in eye-tracking research have been a subject of methodological concern in the field. Orquin and Holmqvist [18] describe critical threats to validity in eye-tracking research, including flexibility in ROI definition and analysis choices that inflate researcher degrees of freedom and affect the interpretability of results. Our analyses empirically demonstrate this concern: slight changes in the ROI margin can flip the significance of effects, a dependency that undermines the reliability of ROI-based inference.

Several studies have developed data-driven ROI generation techniques. For example, applying a threshold to fixation density maps [23] or fixation clustering approaches (e.g., [24,25]) define regions of high fixation density without a priori hypotheses about location. While these algorithms produce objective ROIs based on the data, they remain descriptive: they identify where observers look most frequently, but do not directly test whether fixations in these regions differ between experimental conditions. For instance, in image nine, fixations on the shoes are limited and unlikely to define an ROI.

In contrast, our approach uses a discriminative model to test whether fixation distributions as a whole contain condition-relevant information, operationalised as classifier accuracy. This is conceptually analogous to classification image techniques used in vision science (e.g., [45,46,47]) and to probe DNN properties [30,48], where the goal is to identify features predictive of behavioural decisions or DNN responses.

A key advantage of the classification framework over pixel-wise or cluster-wise comparisons is its parsimony. Traditional voxel- or pixel-wise analyses treat each spatial unit as a separate statistical test, leading to thousands of simultaneous comparisons. Controlling false positives in this setting requires strong multiple-comparisons corrections or non-parametric cluster-based solutions (e.g., [49]), which rely on spatial smoothing, resampling, and relatively large samples to achieve stable inference. While these approaches are well-suited to detecting spatially consistent effects at the group level, they can lose sensitivity when gaze data are sparse, heterogeneous across participants, or weakly aligned in space. In contrast, our decoding-based approach avoids mass-univariate testing altogether by assessing condition information at the level of single fixations. In fact, by training a single classifier per image to predict conditions from individual distributions, we reduce the inferential problem to a single statistical test per image. Reverse correlation then visualises condition-diagnostic spatial patterns without additional inferential testing at every location, sidestepping the need for explicit multiple-comparisons corrections and facilitating clear interpretation of spatial differences. Furthermore, this makes it applicable to data from a single participant or a small number of aggregated participants and robust to spatially distributed effects. In fact, we treat our participants as a meta-observer, pooling their data to obtain approximately 7× the number of trials of a single participant (i.e., ~700 trials in total). Our modelling framework operates directly at the level of trials and does not perform statistical inference on the “average participant.” Instead, it estimates sampling statistics from the aggregated fixation dataset. Importantly, the task duration per participant was relatively short: 100 trials at 2 s per image correspond to approximately 200 s of stimulus presentation time, resulting in a session lasting less than 15 min, including calibration and briefing intervals. Thus, in principle, a single participant could complete the full set of ~700 trials within a feasible session. This means that our approach is applicable at the level of an individual observer. This is important given recent evidence that fixation strategies show stable and systematic individual differences [50], suggesting moving beyond statistical inference on the average observer.

In our approach, it is not possible to treat images as random factors in the conventional sense, because the network explicitly learns image-specific patterns. In fact, we trained one network per image. The goal of this approach is therefore, to some extent, different from the typical random-effects framework used in ROI eye-tracking studies. Our approach allows to make inferences at the level of individual stimuli rather than aiming to generalise across a population of images. Importantly, this design enabled us to observe stimulus-specific idiosyncrasies that could not have been predicted a priori. We believe such an exploratory approach is crucial for generating new, testable hypotheses.

Other machine learning-based approaches have used resampling or classification logic to test hypotheses about gaze behaviour. For example, earlier work used linear classification on eye movement features to assess task effects in scan paths (e.g., [14]). Bootstrap and resampling analyses have been used to assess task-dependent fixation differences (e.g., [12,13]). These frameworks share with our method the idea of using data-driven inference to evaluate task effects but depend on predefined feature extraction as input to the machine learning model. In contrast, our approach does not require manually defined features; instead, it uses a deep network to learn discriminative spatial patterns from fixation data. A crucial advance in predicting fixation distributions with machine learning has been achieved by fine-tuning a DNN pre-trained for object classification [51]. Such a network, in fact, preserves object knowledge, which is critical for predicting visual saliency [38]. While this approach fundamentally differs from ours—since it does not examine differences between experimental conditions but instead aims to predict fixation allocation—a similar strategy could potentially be applied to our purposes, namely, fine-tuning a pre-trained network to detect condition-specific effects. However, this would likely require substantially more data, and future research will need to explore the feasibility and effectiveness of this approach in our specific context.

We included all collected data in the analyses and did not attempt to reduce the number of trials or participants, as systematically testing the lower limits of the approach (e.g., through subsampling observers or trials) was beyond the scope of the present study.

However, our method does not only work with very large datasets. In the current experiment, each image was presented for 2 s, yielding on average approximately 9 fixations per trial (consistent with an expected rate of 3–5 fixations per second: e.g., [52]). The dataset comprises seven participants, each completing five repetitions per condition—amounting to a relatively modest number of fixations overall. Despite this limited data volume, the approach produced stable results. The full dataset is publicly available, also enabling others to directly test the limits of the method if required.

For simplicity, we tracked only the right eye, as we assumed that participants were healthy and that both eyes fixated on the same location. This approach is consistent with previous studies (e.g., [38,39]). However, although we do not expect systematic differences between eyes, tracking both eyes and averaging their signals could have reduced measurement noise [53].

Beyond methodological contributions, our data reveal important behavioural phenomena. One consistent observation was that the presence of sound induced disengagement from faces and central regions of the image—areas that are otherwise highly salient in scene perception. This aligns with the substantial literature showing a default bias for central fixation and faces in free viewing [37,54], but importantly shows that auditory context can systematically alter this bias.

For instance, in image 1, the ROI analysis failed to detect condition differences, but the classifier and associated reverse-correlation maps revealed that participants in the sound condition fixated less on the face and central image area, and more on lateral regions. Similarly, we found that participants’ first fixations were biassed toward the image border closest to the starting position of the fixation cross, which was always randomly placed on the left or right of the image. This is consistent with previous research showing start-position effects on early fixations [42,55]. For image 1, the bias appeared stronger when the sound was present, likely because the auditory cue reduced the salience of the centrally located face and facilitated disengagement from it. This suggests an exploratory gaze strategy in the presence of congruent sounds, potentially aimed at locating the auditory source through spatial sampling rather than relying on default salient features. In image 9, fixation differences emerged not only at the sound-emitting shoe but also in a systematic shift in gaze away from the face toward the body, indicating gaze strategies that simultaneously monitor multiple task-relevant regions such as the sound source and the figure producing it. These distributed strategies are consistent with ideas that gaze deployment optimises perceptual efficiency by balancing sampling across locations relevant to the task [22].

Our findings extend the growing evidence that gaze behaviour is shaped by an interaction of bottom-up saliency and top-down task demands. Early models of visual saliency emphasised image features such as intensity and contrast as determinants of fixation locations. However, subsequent work has shown that cognitive instructions and task demands strongly modulate gaze allocation (e.g., task effects in scene viewing, [6,7,56]). Multisensory influences on gaze distribution observed here suggest that saliency in one modality (auditory) can reshape visual saliency landscapes in a way that is distributed and not confined to local object features, consistent with our recent work on cross-modal effects on visual saliency [16].

Although the present classification approach captures robust spatial differences between conditions, future work could extend this framework to investigate temporal dynamics of gaze allocation and interactions with low-level image features. Additionally, integrating classifier outcomes with behavioural performance or neural measures could yield richer models of multisensory attention.

We present a general analytical framework that is not specific to particular stimuli, experimental variables, item characteristics or tasks, but can in principle be applied to any image-based eye-tracking dataset. Importantly, our results also suggest that the approach does not require large datasets to detect systematic differences because even with a relatively limited number of participants and trials, the DNN was able to identify reliable patterns. While replication across different image-based eye-tracking contexts will need to be demonstrated empirically, the methodological structure of this approach is general and should extend naturally to other studies.

This design is well-suited to studies with a limited number of images, as in the present case, as it allows to investigate stimulus-specific differences. However, it is also well-suited to studies with large stimulus sets. In the latter case, to treat images as a random factor, one could compute performance metrics (e.g., classification accuracy) separately for each image and then conduct statistical testing on these image-level averages (for example, testing accuracy against chance with image entered as a random factor). Importantly, training relatively simple networks is fast with current hardware. Therefore, even when many images are included, fitting separate networks per image and deriving image-level statistics is practically feasible.

## 5. Conclusions

This study highlights a methodological advance in eye-tracking analysis, demonstrating the advantages of a data-driven, classification-based framework over traditional ROI approaches. Our results show that conventional ROI analyses can be highly sensitive to arbitrary parameter choices—small adjustments to ROI size or placement can dramatically alter statistical outcomes. Importantly, because the classification framework is not constrained by pre-defined hypotheses about where effects should occur, it accommodates exploratory investigations and can reveal unexpected, stimulus-specific patterns that traditional ROI methods might miss.

By training a discriminative model to predict experimental condition from single-fixation distributions, our approach reduces the inferential problem to a single, well-defined test per image, avoiding multiple comparisons (e.g., per pixel). Reverse correlation then allows direct visualisation of condition-diagnostic spatial patterns, enabling clear interpretation of effects without introducing additional statistical assumptions. Crucially, this framework operates at the trial level rather than on participant averages, allowing analyses at the level of individual observers and enabling the study of systematic individual differences in fixation strategies.

Our results demonstrate that a data-driven classification approach provides a reliable, objective, and interpretable alternative to traditional ROI analyses and clustering methods. By focusing directly on condition-diagnostic gaze patterns rather than merely on regions that attract a high number of fixations, this method both avoids subjective parameter choices and reveals spatial gaze effects that would otherwise remain undetected. Its hypothesis-free nature makes it particularly suitable for exploratory studies, offering a reliable alternative to traditional analytic pipelines.

Beyond these methodological contributions, our data also reveal meaningful behavioural phenomena, specifically, the presence of congruent sound-induced disengagement from faces and central regions of the image—areas that are otherwise highly salient in natural scene viewing.

## Figures and Tables

**Table 1:** Bootstrap analysis.

Chance Accuracy	0.0025 Quantile Accuracy	0.9975 Quantile Accuracy
0.4484	0.5490	0.6180
0.5042	0.4030	0.4800
0.5499	0.4060	0.4740
0.4075	0.5640	0.6390
0.4903	0.5210	0.6060
0.5501	0.4480	0.5450
0.4483	0.4800	0.5540
0.5215	0.4720	0.5440
0.4538	0.5790	0.6410
0.5383	0.4270	0.4990

## Data Availability

Data and MATLAB code are available on GitHub: https://github.com/matteo-toscani-24-01-1985/A-data-driven-approach-for-comparing-gaze-allocation-across-conditions.git (accessed on 12 March 2026).
